# Protein structure prediction with local adjust tabu search algorithm

**DOI:** 10.1186/1471-2105-15-S15-S1

**Published:** 2014-12-03

**Authors:** Xiaoli Lin, Xiaolong Zhang, Fengli zhou

**Affiliations:** 1 Hubei Key Laboratory of Intelligent Information Processing and Real-time Industrial System, School of Computer Science and Technology, Wuhan University of Science and Technology, Wuhan 430065, P.R.China; 2 Information and Engineering Department of City College, Wuhan University of Science and Technology, Wuhan, 430065, P.R.China

## Abstract

**Background:**

Protein folding structure prediction is one of the most challenging problems in the bioinformatics domain. Because of the complexity of the realistic protein structure, the simplified structure model and the computational method should be adopted in the research. The AB off-lattice model is one of the simplification models, which only considers two classes of amino acids, hydrophobic (A) residues and hydrophilic (B) residues.

**Results:**

The main work of this paper is to discuss how to optimize the lowest energy configurations in 2D off-lattice model and 3D off-lattice model by using Fibonacci sequences and real protein sequences. In order to avoid falling into local minimum and faster convergence to the global minimum, we introduce a novel method (SATS) to the protein structure problem, which combines simulated annealing algorithm and tabu search algorithm. Various strategies, such as the new encoding strategy, the adaptive neighborhood generation strategy and the local adjustment strategy, are adopted successfully for high-speed searching the optimal conformation corresponds to the lowest energy of the protein sequences. Experimental results show that some of the results obtained by the improved SATS are better than those reported in previous literatures, and we can sure that the lowest energy folding state for short Fibonacci sequences have been found.

**Conclusions:**

Although the off-lattice models is not very realistic, they can reflect some important characteristics of the realistic protein. It can be found that 3D off-lattice model is more like native folding structure of the realistic protein than 2D off-lattice model. In addition, compared with some previous researches, the proposed hybrid algorithm can more effectively and more quickly search the spatial folding structure of a protein chain.

## Background

The understanding of molecular conformations is one of the crucial issues in computational biology. The incorrect protein folding is associated with illnesses such as Alzheimer's disease, bovine spongiform encephalopathy and Creutzfeldt-Jakob disease. The biological functions of protein are determined by their dimensional folding structures, and their spatial structures are absolutely determined by their primary structures [1]. Traditional experimental methods of determining protein folding structure are expensive, such as X-ray crystallography and NMR spectroscopy. Because of the complexity of realistic protein, it is extremely difficult to make an analysis of the protein folding process.

Due to the polypeptide chain forms such large number of different spatial structure, it is still difficult to search for the global minimum energy conformations of proteins from its sequence of amino acids [2]. Therefore, the most important problem is how to establish a highly simplified but effective model which can reflect the relation between the free energy and tertiary structure of the protein. One of the simplified protein models is the hydrophobic-polar (HP) model which has been widely used to study protein structure and understand protein folding process. The HP-lattice model represents the amino acid chains of a protein using two types of residue, non-polar or hydrophobic (H) residue and polar (P) or hydrophilic residue are on the vertices of a simple cubic lattices[3]. The HP lattice-model abstracts the hydrophobic interaction process in protein folding by reducing a protein to a heteropolymer that represents a predetermined pattern of hydrophobicity in the protein[4]. The non-ploar amino acids are classified as hydrophobic and polar amino acids, which is used to force the formation of a compact hydrophobic core as observed in the real protein [5]. However, the HP lattice-model doesn't reveal all secrets of the protein, despite its simplicity. The main reason lies in that local interactions are neglected in the simplified models, while local interactions might be important for the local structure of the chains [6].

To reflect more realisticly the native attributes of proteins, Stillinger studied a similar AB off-lattice protein model in two dimensions[7]. In AB off-lattice model the 20 amino acids are also reduced to two classes, hydrophobic (A) and hydrophilic (B). For AA, BB and AB pairs respectively, there is an intramolecular mix of strong attraction, weak attraction, and weak repulsion, roughly analogous to the situation of the real proteins[8].The interactions considered in AB off-lattice model include both sequence independent local interactions and the sequence dependent Lennard-Jones term that favours the formation of a hydrophobic core[9]. Irback et al. extended a two dimension (2D) to a three dimension (3D) in the AB off-lattice model, which takes account of the torsional energy implicitly [6].

In recent years, many works were devoted to the optimal conformations with lowest energies in the AB off-lattice model[10-12]. Because searching the whole conformational space of a protein has been proved to be NP-complete problem, it is necessary to introduce the heuristic optimization algorithm, such as the energy landscape paving minimizer (ELP)[9], the genetic tabu search algorithm (GATS)[10], the conformational space annealing (CSA)[13], the pruned-enriched-Rosenbluth method (PERM) [14] and the local adjust genetic algorithm (LAGAA) [15] etc. This paper describes a protein structure prediction method that is based on the AB off-lattice model in two dimension and three dimension, which combines the tabu search algorithm and local adjust simulation annealing. The new improved hybrid algorithm (SATS) is applied to find the spacial conformations with Fibonacci sequences and real proteins.

## Methods

### AB off-lattice model in 2D

**Figure 1:**
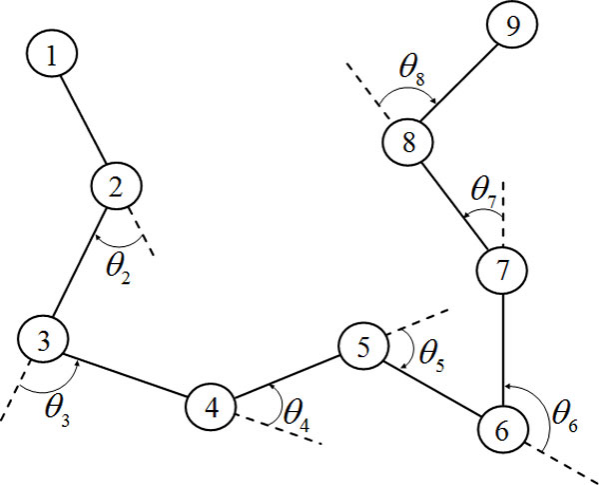
**A schematic diagram of a generic 9-mer in 2D**. The *n*-mer is specified by the *n - *2 angles of bend *θ*_2_,..., *θ_n−_*_1_.

The major difference between AB off-lattice model [7] and HP lattice model [3] is the folding angle of the model. The AB off-lattice model deals with only two types of amino acids, to be called hydrophobic A and hydrophilic B, which can be used to represent 20 kinds of amino acids. They are linked together by rigid unit-length bonds to form linear unoriented polymers that reside in the form of two dimensions. In 2D AB off-lattice model the angle of the two bonds which connect three amino acid residues can change freely, as Figure 1 illustrates [7], the configuration of any *n*-mer is specified by the *n − *2 angles of bend *θ*_2_,..., *θ_n−_*_1 _at each of the nonterminal residues. *θ_i _*= 0 corresponds to linearity of successive bonds.

The AB off-lattice model postulates that two kinds of interactions including the intramolecular potential energy for each molecule: backbone bend potentials *V*_1 _and nonbonded interactions *V*_2_. Residue species along the backbone can be conveniently encoded by a set of binary variables *ξ_i_*. If *ξ_i _*= 1, the *ith *residue is A; if *ξ_i_*=-1, it is B. Hence, the total energy function for any *n*-mer chain is expressed as follows [7]:

(1) $$\text{Φ}=\overset{n-1}{\underset{i=2}{\sum }}V_{1}\left(\theta_{i}\right)+\overset{n-2}{\underset{i=1}{\sum }}\overset{n}{\underset{j=i+2}{\sum }}V_{2}\left(r_{ij},\xi_{i},\xi_{j}\right)$$

Where the distance *r_ij _*between monomer *i *and *j *is denoted as functions of the intervening angles[7]:

(2) $$r_{ij}=\{[\sum_{i=2}^{j-1}cos{\left[\sum_{l=i+1}^{k}\theta_{1}]\right]}^{2}+{\left[\sum_{i=2}^{j-1}sin{\left[\sum_{l=i+1}^{k}\theta_{1}]\right]}^{2}\right\}}^{1/2}$$

The *V*_1 _is a simple trigonometric form:

(3) $$V_{1}\left(\theta_{i}\right)=1/4\left(1-\text{cos}\theta_{i}\right)$$

The *V*_2 _is non-bonded interactions with a species-dependent coefficient:

(4) $$V_{2}\left(r_{ij},\xi_{i},\xi_{j}\right)=4\left(r_{ij}^{-12}-C\left(\xi_{i},\xi_{j}\right)r_{ij}^{-6}\right)$$

where

(5) $$C\left(\xi_{i},\xi_{j}\right)=\left\{\begin{array}{c} +1\;\;\;AA\\ +1/2\;BB\\ -1/2\;AB \end{array}\right)$$

For any *N *-residue protein, the *N − *2 angles of bend should be found when the potential-energy of the 2D AB off-lattice model obtains the minimum energy, which is based on the thermodynamic hypothesis formulated by Anfinsen: the natural structure of a protein in its physiological environment is the one in which the free energy of the whole system is lowest[1]. Thereby, the protein folding problem can be defined:

(6) $$\text{min}\left\{\text{Φ}\left(\theta_{2},...,\theta_{n-1}\right)\right\}\;\;\theta_{i}\varepsilon \left(-\pi ,\pi \right)$$

### AB off-lattice model in 3D

The 3D AB off-lattice model also consists of hydrophobic (A) residues (*σ_i _*= +1) and hydrophilic (B) residues (*σ_i _= −*1), and the energy function is given by [6]

(7) $$E=-k_{1}\overset{N-2}{\underset{i=1}{\sum }}{\hat{b}}_{i}\cdot {\hat{b}}_{i+1}-k_{2}\overset{N-3}{\underset{i=1}{\sum }}{\hat{b}}_{i}\cdot {\hat{b}}_{i+2}+\overset{N-2}{\underset{i=1}{\sum }}\overset{N}{\underset{j=i+2}{\sum }}E_{IJ}\left(r_{ij};\sigma_{i},\sigma_{j}\right)$$

Where ${\hat{b}}_{i}$ is defined as the bond vector between the monomers *i *and *i *+ 1 with unit length:

(8) $$\left\{\begin{array}{c} {\hat{b}}_{i}\cdot {\hat{b}}_{i+1}=\text{cos}\theta_{i}\\ {\hat{b}}_{i}\cdot {\hat{b}}_{i+2}=\text{cos}\alpha_{i} \end{array}\right)$$

**Figure 2:**
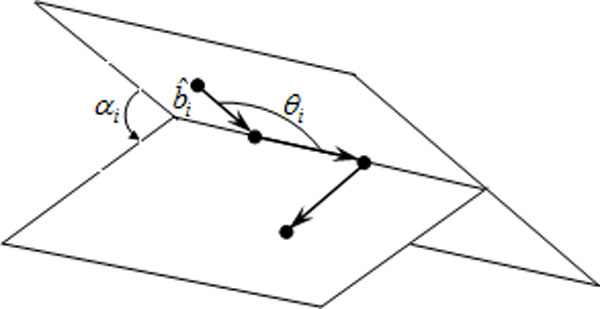
**A schematic diagram of protein folding in 3D**. The *N *-mer can be specified by the *N − *1 bond vectors ${\hat{b}}_{i}$ or by *N − *2 bond angles *θ_i _*and *N − *3 torsional angles *α_i_*.

As Figure 2 illustrates[6], the *N *-residue molecule can be described by the *N − *1 bond vectors ${\hat{b}}_{i}$ or by *N − *2 bond angles *θ_i _*and *N − *3 torsional angles *α_i_*, and these two angles are the degrees of freedom of the 3D off-lattice model.

The Eucledian distance *r_ij _*between sites *i *and *j *is associated with the *N − *2 bond angles and *N − *3 torsional angles. The species-dependent global interactions are given by the Lennard-Jones potential[6]:

(9) $$E_{LJ}\left(r_{ij};\sigma_{i},\sigma_{j}\right)=4C\left(\sigma_{i},\sigma_{j}\right)\left(1/r_{ij}^{12}-1/r_{ij}^{6}\right)$$

Where *σ*_1_, *. . *., *σ_n _*is a binary string:

(10) $$\sigma_{i}=\{\begin{array}{cc} 1 & A\\ -1 & B \end{array}$$

If *σ_i _*= 1, the *ith *reside is A; if *σ_i _= −*1, the *ith *reside is B, and the formation of hydrophobic core depends on the *C(σ_i_, σ_j_*). In addition, the strength of species-independent local interactions is reflected by the parameters *K*_1 _and *K*_2_. The parameter (*K*_1_, *K*_2_) were tested again and again by using different values in [6], and finally Irback found that the spacial structure is more stability when the parameter (*K*_1_, *K*_2_) was set to (*−*1, 0.5).

As same as, the *N − *2 bond angles *θ_i _*and *N − *3 torsional angles could be found by computing the lowest energy of residue sequences. So the protein folding problem of the 3D AB off-lattice model can be specified as:

(11) $$\text{min}\left\{E\left(\theta_{2},...,\theta_{n-1};\alpha_{3},...,\alpha_{n-1}\right)\right\}\;\theta_{i},\alpha_{i}\varepsilon \left(-\pi ,\pi \right)$$

### Improved strategies

Tabu search, created by Glover [16], is a meta-heuristic search method. It can be used for complex mathematical optimization and combinatorial optimization problems. Tabu search uses a local or neighborhood search procedure to iteratively move from one potential solution to an improved solution in the neighborhood until some stopping criterion has been satisfied. Local search procedures often become stuck in poor-scoring areas or areas where scores plateau. In order to avoid these pitfalls and explore regions of the search space that would be left unexplored by other local search procedures, tabu search carefully explores the neighborhood of each solution as the search progresses. The solutions admitted to the new neighborhood, are determined through the use of the memory structures. Adaptive memory helps the search process to avoid local optima and explores the solution space economically and effectively without getting trapped into cycles [17]. These memory structures form what is known as the tabu list, a set of rules and banned solutions used to filter which solutions will be admitted to the neighborhood to be explored by the search. To enhance the efficiency, the following strategies are used in the algorithm for predicting the protein folding structure.

#### Encoding

It is very important for the algorithm how to encode the individual, because the different encoding will affect the effectiveness and performance of searching for the whole spatial structure. The solutions of individual encoding then are often binary coded. This encoding, however, is not well suited for protein folding structure prediction problem. Instead, for an N-residue long chain, the individual can be expressed as $h_{i}=\left\{\theta_{2}^{i},\theta_{3}^{i},\ldots ,\theta_{n-1}^{i}\right\}$ and $h_{i}=\left\{\theta_{2}^{i},\theta_{3}^{i},\ldots ,\theta_{n-1}^{i},\alpha_{3}^{i},\alpha_{4}^{i},\ldots ,\alpha_{n-1}^{i}\right\}$ in the 2D AB off-lattice model and the 3D AB off-lattice model respectively. Encoding in this way is enabled by the fact that the optimization is performed for the protein amino acids chain.

#### Annealing mechanism

Simulated Annealing (SA) [18]is a probabilistic method for the global optimization problem of searching an approximation to the global optimum of a cost function. Just as the cooling process of solid shows, the solid stays in a disorder state at the beginning with a high temperature, and coming to more and more order when the temperature drops lower and lower till to the frozen state [19]. The core mechanism of SA is the Metropolis Criterion which is used to decide whether the new state should be accepted. The acceptance probability function depends on the energy E and temperature T. If the change in energy is negative the new state is accepted. If the change in energy is positive it is accepted by the certain probability given by the Boltzmann factor. That is to say, the good and bad solution both can be accepted with a probability to avoid becoming trapped in a local optimum. Annealing algorithm simulates the process described above, the algorithm starts with a give parameter called start temperature, and terminates when the temperature drops to zero or the global optimized solution is founded. In this paper, the cooling schedule is a simple linear equation which is the same as [20] (*T_i+1 _= σT_i_*, 0 *≤ σ ≤ *1, When *σ *inclines to 1, the temperature declines only slowly).

#### Adaptive neighborhood generation

Neighborhood choosing is a key activity for tabu searching. In order to improve the performance of the algorithm, an optimized neighborhood choosing strategy as the following described is adopted. In our strategy, the neighborhood is related to the current annealing state and initial temperature. With the change of the current annealing state and the decreasing of the temperature, the neighborhood is more and more small. To illustrate the relation between annealing state and neighborhood, a linearity combination can be defined:

(12) δNeibor=scale*CurState/InitState

Where *scale *represents the original neighborhood of the tabu search, *CurState *is current annealing state, and *InitState *represents the initial annealing state. So the neighborhood gradually narrows in the process of annealing.

#### Local adjustment strategy

**Figure 3:**
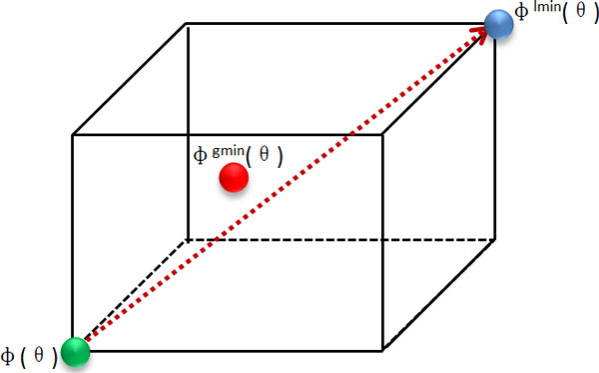
**A schematic diagram of local adjustment**.

Figure 3 presents the schematic diagram of local adjustment for the tetramer. If there is a local minimum solution found, how to get a further optimize solution depend on it, is a worth considering problem. In many cases, the global minimum solution is near to the local minimum solution. Base on this conclusion, a local adjust strategy is adopted to enhance the performance of the algorithm. Give *φ^lmin^ (θ*), *φ^gmin^ (θ*) and *φ(θ*), standing for a gotten local minimum solution, the global minimum solution to be founded and a current solution to generate off-spring. So the vector *φ^lmin^ (θ*) - *φ(θ*) shows the direction for the off-spring of *φ(θ*) to move towards. As the off-spring moves, the possibility of finding global minimum solution will increase. Aiming to make a simple description, we define:

(13) $$\varphi \left(\theta \right)=f\left(\theta_{x},\theta_{y},\theta_{z}\right)$$

The vector *φ^lmin^ (θ) − φ(θ*) constrains the off-spring of *φ(θ*) in a cube area, if *φ^gmin^ (θ*) is lying in this area, the possibility of finding it will be increasing.

#### The algorithm

The SATS algorithm is based on the AB off-lattice Model. Just as the followed process illustrates, the algorithm generates a hypotheses list by using the same initial conformation mechanism in [21] (The idea is as follows: Pick out all A-monomers and place them in certain spots in the space, and all B-monomers wrap the hydrophobic core.), and calculates every individual's energy of the list by the AB off-lattice model and stores the individual with the best energy as a temp best solution. Then, start to descend the temperature, during this period, a new list with individuals in a small scale around the individuals of the hypotheses list is produced as neighborhood list. After calculate out the individual energy, the neighborhood list is rear-ranged by the energy of the individual. Select several top individuals of the neighbor-hood list as candidates and use deprecated principle to judge whether to add it to the tabu list or not. As the tabu list refreshed, the local adjust principle is adopted to optimize the elements of the list aimed to find the possible best solutions. If the current temperature is lower than the given stop condition, terminate the algorithm and output the founded best solution. The steps of SATS algorithm are as following.

SATS Begin

Parameter:

neighbourhood_size

candidate_size

initial_temperature

end_temperature

descend_rate

Process:

Step 1: Create hypotheses using the initial conformation mechanism;

Step 2: Generate neighborhood solutions;

Step 3: Calculate the energy value by the off-lattice Model;

Step 4: Select candidate solutions from neighborhood;

Step 5: Use deprecated principle to accept the solution and add it to the Tabu List;

Step 6: Apply the local adjustment strategy;

Step 7: Descend the temperature, if the temperature is greater than the end temperture then go to Step 2;

*Step 8: Output the Result*.

*SATS End*.

## Results and discussion

The SATS algorithm has been implemented with Python in Windows 7. There are two parts of experiment for searching the optimum energy conformation in the AB off-lattice model.

### Results for Fibonacci sequences

In the experiments of protein folding structure prediction, the Fibonacci sequences are usually selected as the experiment data to test the performance of the optimization algorithm. The Fibonacci sequences can be defined:

(14) $$s\left(n\right)=\left\{\begin{array}{c} A\;\;\;\;\;\;\;\;n=0\\ B\;\;\;\;\;\;\;\;n=1\\ s\left(n-2\right)+s\left(n-1\right)\;n>1 \end{array}\right)$$

**Figure 4:**
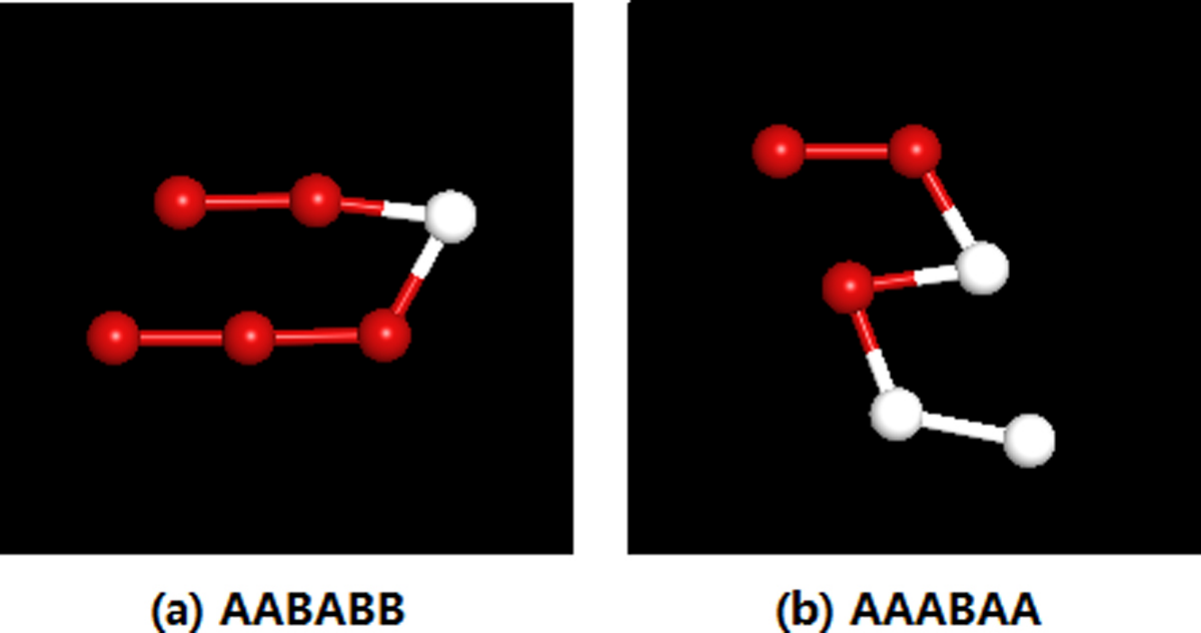
**The schematic diagram of helix and sheet**. (a)The lowest energy of AABABB is -1.36198.(b) The lowest energy of AAABAA is -3.69750.).

For comparison, the first part adopts the same short Fibonacci sequences with 3 *<= N <*= 5 in Ref. [7]. Table 1 lists the lowest energy values of thirty-six short Fibonacci sequences calculated by our SATS, and we believe that these spacial conformation are the ground states, which are entirely identical to those of Stillinger [7]. We also use two same test sequences in [8] to obtain their minimal energies and secondary structures, as Figure 4 illustrates. It is seen from Figure 4 that "AABABB" belongs to *α*-helix and "AAABAA" belongs to *β*-sheet.

**Table 1:** The minimum energies obtained by SATS for the short Fibonacci sequences.

SEQUENCE	ENERGY	SEQUENCE	ENERGY
AAA	-0.65821	AAAAA	-2.84828
AAB	0.03223	AAAAB	-1.58944
ABA	-0.65821	AAABA	-2.44493
ABB	0.03223	AAABB	-0.54688
BAB	-0.03027	AABAA	-2.53170
BBB	-0.03027	AABAB	-1.34774
		AABBA	-0.92662
AAAA	-1.67633	AABBB	0.04017
AAAB	-0.58527	ABAAB	-1.37647
AABA	-1.45098	ABABA	-2.22020
AABB	0.06720	ABABB	-0.61680
ABAB	0.64938	ABBAB	-0.00565
ABBA	-0.03617	ABBBA	-0.39804
ABBB	0.00470	ABBBB	-0.06596
BAAB	0.06172	BAAAB	-0.52108
BABB	-0.00078	BAABB	0.09621
BBBB	0.13974	BABAB	-0.64803
		BABBB	-0.18266
		BBABB	-0.24020
		BBBBB	-0.45266

Then we test our algorithm on four Finbonacci sequences with 13 *≤ n ≤ *55. Firstly, let us discuss the situation of the 2D AB off-lattice model. The methods used to search lowest energy states include neural networks [7], the pruned-enriched-Rosenbluth method [14], the particle swarm optimization (*E_EPSO_*) [12]. Table 2 lists the lowest energies obtained by our SATS along with those of above three methods for comparison. Of course, with the increase of the sequence length, the search time will longer. It is obvious that our lowest energy values are agree with those of EPSO for *n *= 13 and *n *= 21. For other cases, our energy values are lower than the energy values obtained by other three methods, especially for the sequence with *n *= 55, the result of SATS is obviously improved.

**Table 2:** The minimum energies obtained by different algorithm for Fibonacci sequences with 13 *≤ N ≤ *55 in 2D.

N	SEQUENCE	*E_min_*	*E_perm_*	*E_EPSO_*	*E_SAT S_*
13	ABBABBABABBAB	-3.224	-3.217	-3.294	-3.294
21	BABABBABABBAB	-5.288	-5.750	-6.198	-6.198
	BABABBAB				
34	ABBABBABABBAB	-8.975	-9.220	-9.834	-10.707
	BABABBABABBABBABABBAB				
55	BABABBABABBABBABABBABAB	-14.409	-14.905	-16.447	-18.467
	BABBABABBABBABABBABABBABBABABBAB				

**Figure 5:**
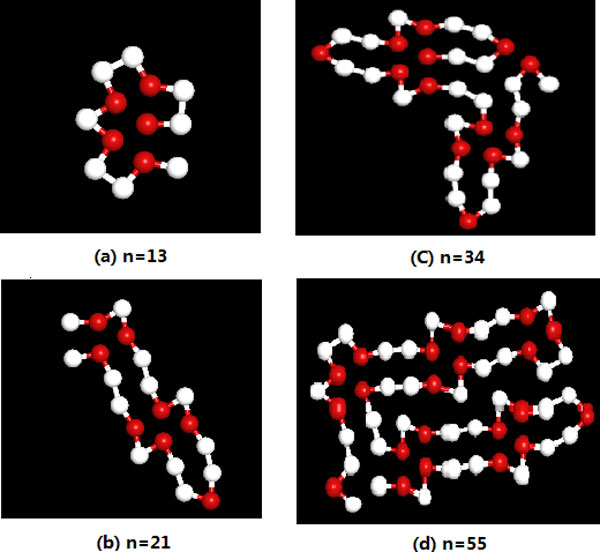
**The 2D lowest energy conformations of the four Fibonacci sequences obtained by SATS algorithm**. (a) *n *= 13; (b) *n *= 21; (c) *n *= 34; (d) *n *= 55. (The red balls represent hydrophobic A monomers, white balls represent hydrophilic B monomers.

In order to further analyze the effect of the SATS algorithm, we also give the lowest energy conformations of four Finbonacci sequences in Figure 5. The hydrophobic (A) is represented by the red ball, and the hydrophilic (B) is represented by the while ball. As is known to all, a hydrophobic cores always are surrounded by hydrophilic residues for a realistic protein chain. It is observed from four conformations that *n *= 13 forms the single hydrophobic core, while other three conformations only form the particle clusters. This shows that the 2D AB off-lattice model can show some important attributes of the real protein to certain extent, and it still should be improved.

**Figure 6:**
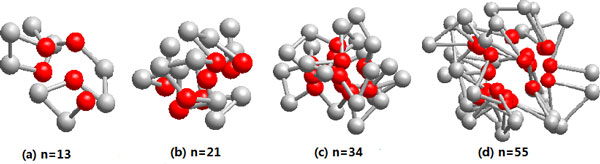
**The 3D lowest energy conformations for the four Fibonacci sequences obtained by SATS**. (a) *n *= 13; (b) *n *= 21; (c) *n *= 34; (d) *n *= 55. (The red balls represent hydrophobic A monomers, white balls represent hydrophilic B monomers.

In the case of 3D AB off-lattice model, it also shows that the results obtained with SATS are better than the results of the previous algorithms. The methods used to search lowest energy states in 3D off-lattice model include the annealing contour Monte Carlo (ACMC) algorithm [22], the energy landscape paving minimizer ELP [9], the conformational space annealing (CSA) algorithm [13] and the local adjust genetic algorithm studied by us in [23]. Table 3 lists the lowest energies obtained by different methods. For *n *= 13, the energy value of SATS is agree with that of ACMC, but is lower than energy values obtained by other three method. For *n *= 21, our energy value is agree with those of ELP and LAGA, but is slightly lower than that of CSA and much lower than that of ACMC. For *n *= 34 and *n *= 55, our results are the lowest energy in all results obtained by different methods, which shows that SATS has better performance for long sequence. Similar to 2D prediction, Figure 6 depicts the 3D lowest energy conformations with the same way in [21] (Where the hydrophobic (A) is denoted by the red ball, the hydrophilic (B) is denoted by the grey ball). Each of four sequences can fold into a single hydrophobic core flanked by the hydrophilic residues, compared with 2D off-lattice model, the 3D AB off-lattice model is more approach to realistic protein folding structure.

**Table 3:** The minimum energies obtained by different algorithm for Fibonacci sequences with 13 *≤ N ≤ *55 in 3D.

N	SEQUENCE	*E_ELP_*	*E_ACMC_*	*E_CSA_*	*E_LAGA_*	*E_SATS_*
13	ABBABBABABBAB	-26.498	-26.507	-26.471	-26.498	-26.507
21	BABABBABABBAB	-52.917	-51.757	-52.787	-52.917	-52.917
	BABABBAB					
34	ABBABBABABBABBABABBAB	-92.746	-94.043	-97.732	-98.765	-99.876
	ABBABBABABBAB					
55	BABABBABABBABBABABBAB	-172.696	-154.505	-173.980	-176.542	-178.986
	ABBABBABABBABBABABBAB					
	ABBABBABABBAB					

**Figure 7:**
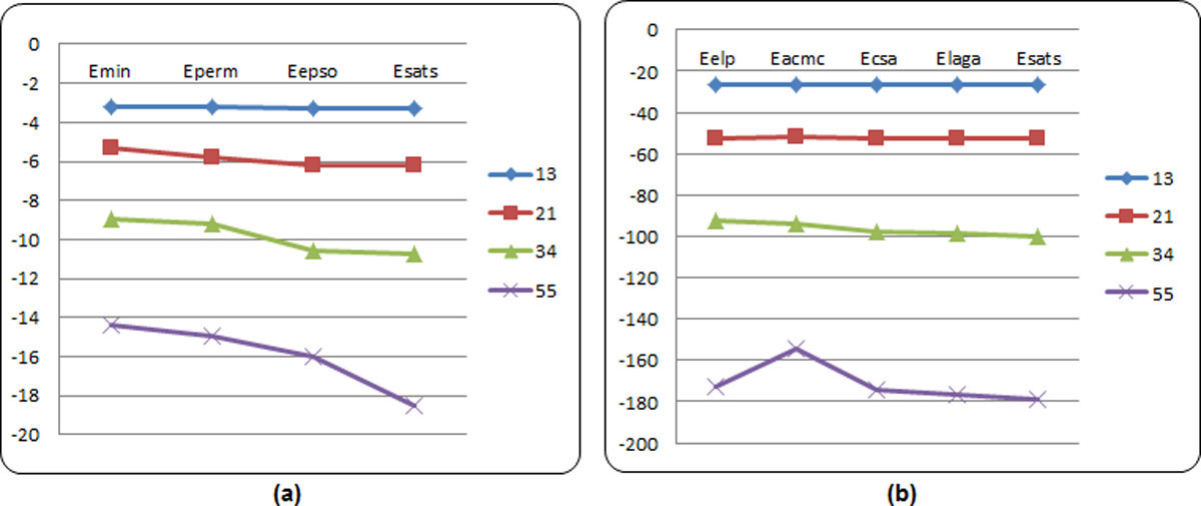
**The energy curves obtained by different algorithms**. (a) The energy curves in the 2D model;(b) The energy curves in the 3D model.

The curves in the Figure 7 are about the minimum energies of 2-dimensional model and 3-dimensional model obtained by different algorithm. From Figure 7, we can find that our methods show much better performance with increasing sequence length.

### Results for real protein sequences

**Figure 8:**
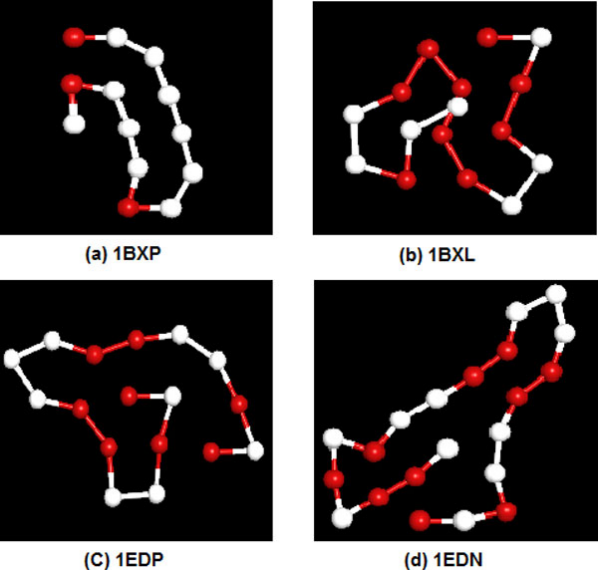
**The lowest energy conformation for the four real protein sequences obtained by SATS**. (a)1BXP; (b) 1BXL; (c) 1EDP; (d) 1EDN. (The red balls represent hydrophobic A monomers, the white balls represent hydrophilic B monomers.

The second part deals with some real protein sequences from the Protein Data Bank (PDB). The K-D method [24] is adopted to distinguish between hydrophobic residues and hydro-philic residues. In short, *I, V, L, P, C, M, A *and *G *are belong to hydrophobic residues (A), while *D, E, F, H, K, N, Q, R, S, T, W *and *Y *are belong to hydrophilic residues (B). Table 4 lists the same four short real protein studied by Ref.[12]. Table 5 shows the minimum energy obtained by four different algorithms. The results of SATS are lower than those of other three algorithms, especially for *1EDN*. This shows that SATS is effective to predict the folding structure of the real protein. Figure 8 shows the lowest energy configurations of four real protein sequence obtained by the SATS method, corresponding to the lowest energy values in Table 5. It can be seen from the Figure 8, the configurations of the *1EDP *and *1BXL *form the hydrophobic core respectively, the other two protein sequences only formed several particle clusters.

**Table 4:** The four short sequences of real protein.

PDB ID	SEQUENCE
1BXP	MRYYESSLKSYPD
1BXL	GQVGRQLAIIGDDINR
1EDP	CSCSSLMDKECVYFCHL
1EDN	CSCSSLMDKECVYFCHLDIIW

**Table 5:** The lowest optimum energies of the short real protein sequences.

PDB ID	*E_GAA_*	*E_LAGAA_*	*E_EPSO_*	*E_SATS_*
1BXP	-2.24484	-2.24484	-4.392713	-4.42913
1BXL	-8.74685	-8.81260	-8.847081	-8.907082
1EDP	-5.60713	-6.64530	-10.06692	-11.06572
1EDN	-7.09609	-7.81925	-11.13420	-13.15426

**Figure 9:**
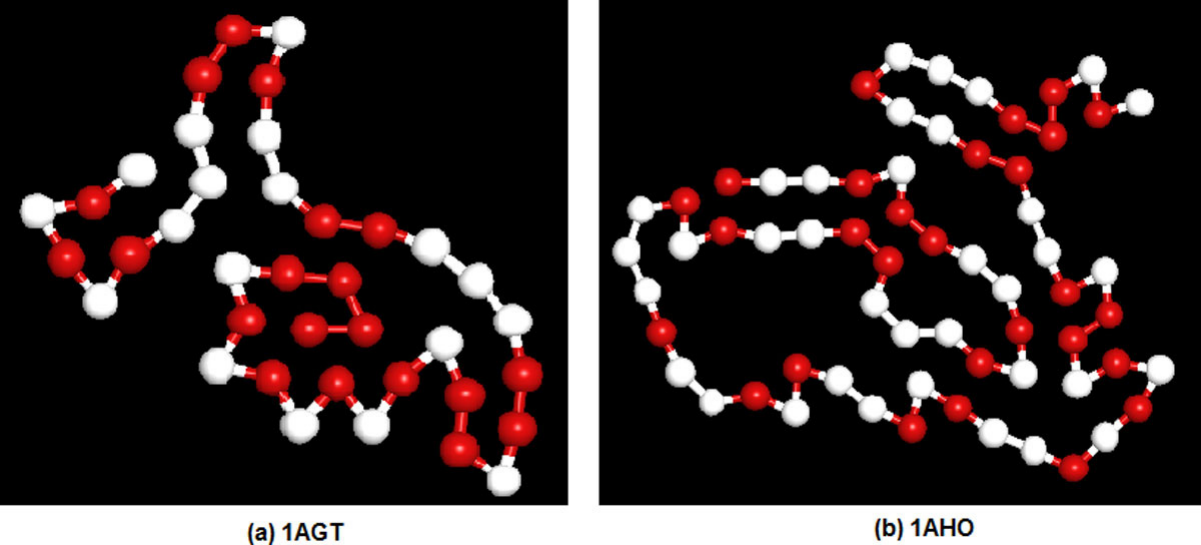
**The lowest energy conformation of 1AGT and 1AHO**. (The red balls represent hydrophobic A monomers, the white balls represent hydrophilic B monomers.).

Table 6 lists the same two long real proteins of *1AGT *and *1AHO *studied by Ref.[11]. For *1AHO*, the result of SATS is much better than those of PSO [11] and SA [25]. For *1AGT*, the result is better than the result of SA while is slightly worse than the result of PSO. Figure 9 shows the information about sequence of two real proteins from PDB, and depicts the lowest energy conformations obtained by SATS. In these two conformations, the hydrophobic monomers also form several clusters of hydrophobic residues. This experiment also demonstrates the AB off-lattice model can reflect some characteristics of the real protein.

**Table 6:** The lowest optimum energies of the two long real protein sequences.

PDB ID	*E_PSO_*	*E_SA_*	*E_GAA_*	*E_SATS_*
1AGT	-19.61686	-17.36282	-19.07243	-19.50661
1AHO	-15.19110	-14.96127	-17.93291	-18.37535

## Conclusions

This paper has shown that protein folding conformation based on only anfinsen's thermodynamic hypothesis can be feasible by SATS method which combines simulated annealing algorithm and tabu search algorithm. In order to verify the efficiency of the algorithm, 2D off-lattice model and 3D off-lattice model are both adopted by using Fibonacci sequences and real protein sequences respectively. In addition, local adjust strategy is used to improved the accuracy and speed of searching the protein native state. It is obvious that some of our results for lowest energy are better than those of other methods. Therefore, SATS is more effective in solving the protein folding structure problem. In the future, the one of most important work is how to make the algorithm more effective and accuracy for real protein sequence prediction in 3D space. Besides, the AB off-lattice model only considers two kinds of residues and two kinds of interaction energy, so it cannot reflect more important properties of the real protein. Therefore, we should study other models to explore the more interaction energy of protein amino acids.

## Competing interests

The authors declare that they have no competing interests.

## Authors' contributions

XL designed the algorithm and analyzed the experimental results. XZ participated in the implementation of algorithm, and did the experiments with the given data. XL and FL wrote the original version of the paper. XZ helped rewriting the paper based on the original version. XL and XZ provided many useful insights on protein modeling. All authors agreed on the content of the paper.

## Declarations

Publication charges for this article have been funded by the Project (No. 61273225) from National Natural Science Foundation of China.

This article has been published as part of *BMC Bioinformatics *Volume 15 Supplement 15, 2014: Proceedings of the 2013 International Conference on Intelligent Computing (ICIC 2013). The full contents of the supplement are available online at http://www.biomedcentral.com/bmcbioinformatics/supplements/15/S15.
